# Differences in Dual Task Performance After Robotic Upper Extremity Rehabilitation in Hemiplegic Stroke Patients

**DOI:** 10.3389/fneur.2021.771185

**Published:** 2021-12-09

**Authors:** Kuem Ju Lee, Gyulee Park, Joon-Ho Shin

**Affiliations:** ^1^Department of Rehabilitation and Assistive Technology, Korea National Rehabilitation Research Institute, Seoul, South Korea; ^2^Translational Research Center for Rehabilitation Robots, National Rehabilitation Center, Ministry of Health and Welfare, Seoul, South Korea; ^3^Department of Neurorehabilitation, Korea National Rehabilitation Hospital, Seoul, South Korea

**Keywords:** cognitive-motor interference, dual-task, motor skills, robotic rehabilitation, stroke

## Abstract

**Background:** Cognitive–motor interference is a phenomenon in which the concomitant performance of cognitive and motor tasks results in poorer performance than the isolated performance of these tasks. We aimed to evaluate changes in dual-task performance after robotic upper extremity rehabilitation in patients with stroke-induced hemiplegia.

**Methods:** This prospective study included patients with left upper limb weakness secondary to middle cerebral artery stroke who visited a rehabilitation hospital. Participants performed a total of 640 robot-assisted planar reaching movements during a therapist-supervised robotic intervention that was conducted five times a week for 4 weeks. Cognitive and motor performance was separately evaluated in single- and dual-task conditions. The digit span test and Controlled Oral Word Association Test (COWAT) were used to assess cognitive performance, whereas motor performance was evaluated through kinematic assessment of the motor task.

**Results:** In single-task conditions, motor performance showed significant improvement after robotic rehabilitation, as did the scores of the COWAT subdomains of animal naming (*p* < 0.001), supermarket item naming (*p* < 0.06), and phonemes *(p* < 0.05). In dual-task conditions, all motor task performance variables except mean velocity showed improvement after robotic rehabilitation. The type of cognitive task did not affect the dual-task effect, and there were no significant differences in the dual-task effects of motor, cognitive, or the sum of motor and cognitive performance after robotic rehabilitation.

**Conclusion:** Post-stroke robotic rehabilitation has different effects on motor and cognitive function, with more consistent effects on motor function than on cognitive function. Although motor and cognitive performance improved after robotic rehabilitation, there were no changes in the corresponding dual-task effects.

## Introduction

Most people commonly experience situations in which they need to perform dual tasks, such as walking while talking with others, or choosing items in the market while calling on their mobile phones. Thus, the ability to perform dual tasks simultaneously is a necessary skill in daily life. Cognitive–motor interference (CMI) is manifested as dual-task effects (DTEs), in which the concomitant performance of both cognitive and motor tasks is reduced as compared to when performing isolated cognitive or motor tasks (1, 2). CMI occurs because performance capacity, which is comprised of both cognitive and motor performance, is limited. This phenomenon is particularly pronounced among stroke patients because of the diminished capacity for dual tasks secondary to stroke (3, 4).

Most studies on CMI among stroke patients have reported lower extremity performance, such as gait and posture control (1, 5–7). CMI in upper limb performance is also important, as most stroke patients with hemiplegia after a stroke have difficulty using the upper limb. Recently, CMI has also been reported in the upper limbs of stroke patients (8, 9). We have also investigated upper extremity motor CMI during various cognitive tasks, in participants with stroke who have undergone robotic rehabilitation (10). However, most studies on CMI, including our previous studies, have focused on only one aspect of cognitive or motor performance. It has been recommended that changes across absolute and relative dual-task performance and the interaction between cognition and motor performance be investigated, to consider treatment effects on overall dual-task performance and to improve understanding of CMI (11, 12). Therefore, it is necessary to explore longitudinal changes in CMI considering the concomitant reciprocal interaction between cognitive and motor performance, in order to assess treatment effects.

Modality transfer, in which training for a specific task improved learning of a novel task, has been reported (13). In particular, physical training has shown modality transfer on various aspects of cognitive function (14). Therefore, we hypothesized that rehabilitation focusing on motor function might improve motor as well as cognitive performance, and that CMI may be changed when using a different strategy between cognitive and motor performance. Therefore, we applied robotic rehabilitation, focusing on upper limb motor function, and explored concomitant changes in motor and cognitive performance, and the concomitant DTEs on both motor and cognitive performance, in order to gain insight regarding overall dual-task performance.

## Methods

### Participants

Participants were consecutively selected from the inpatient department of our rehabilitation center. The inclusion criteria for study participation were as follows: (1) left upper limb weakness secondary to a first unilateral middle cerebral artery stroke, affecting the right hemisphere, as evidenced by brain imaging or medical records; (2) age 18–65 years; and (3) a score ≥ 25 on the Mini-Mental State Examination (MMSE) (15). The exclusion criteria were as follows: (1) orthopedic or neurological conditions other than stroke; (2) aphasia, which would prevent language-related cognitive tasks in the present study; and (3) visual or auditory problems that prevented participation in the study protocol. Based on these criteria, of the 53 participants admitted to our rehabilitation center, 13 participants in the chronic phase of a first-ever stroke were selected for this study.

This study was approved by the Ethics Committee of the Institutional Review Board of our center, and all participants provided informed written consent before enrollment, in accordance with the Declaration of Helsinki.

### Tasks

Motor performance was assessed using a kinematic assessment from the point-to-point task embedded in InMotion 2. We collected data on motor performance variables, including smoothness (SM), mean velocity (MV), path error (PE), and reach error (RE). For SM and MV, a higher value indicates better performance, whereas for PE and RE, a lower value indicates better performance. Detailed explanations of these variables have been described in a previous study (10).

Cognitive performance was measured with two different cognitive task types: (1) the digit span test (DST) and (2) the Controlled Oral Word Association Test (COWAT) from the Seoul Neuropsychological Screening Battery (SNSB-II) (16). The DST, which consists of a forward (DST-for) and backward test (DST-back), was used to assess attention or the central executive component of working memory. The COWAT was a measure of fluency in meaning (animal names: COWAT-animal, supermarket item naming: COWAT-supermarket, and text phoneme naming: words that start with Korean character ¬, °, ⋏; COWAT-phonemic), indicating language proficiency and executive function. In addition to the raw score, we used *z*-scores that were standardized according to the age and educational criteria of the SNSB-II based on a nationwide sample (1,100 people) (16). The order of cognitive tasks was randomized across participants.

### Interventions

For the robotic intervention, an InMotion 2 (Interactive Motion Technologies Inc., Watertown, MA, USA), which was specifically designed for upper limb rehabilitation, was used (2), as described previously (10).

Participants sat in a chair with their trunk restrained to minimize compensatory movement, and their affected arm was placed in an arm support attached to the handle of the robotic arm. With a computer monitor presenting visual feedback in front of the participant, the therapist guided the participant to hold the robot handle and direct the patient to complete moving the handle to one of eight equally spaced points on the perimeter of a 14-cm radius circle from the central object, to complete a 640 planar point-to-point reach movement. The therapist instructed and assisted the patient from the front of the patient's unaffected side.

The task was performed at a comfortable speed without time limitation and the robotic intervention was conducted five times a week for 4 weeks under the supervision of a physical therapist. It has been reported that a large amount of high-dose intensive training and repeated execution of specialized functional tasks play an effective role to activate neural plasticity through robotic intervention (17). In addition, because the functional levels of the upper extremities of the subjects in this study were similar, the number of repetitions was controlled rather than the time of robotic intervention.

### Outcome Measures

**Figure 1:**
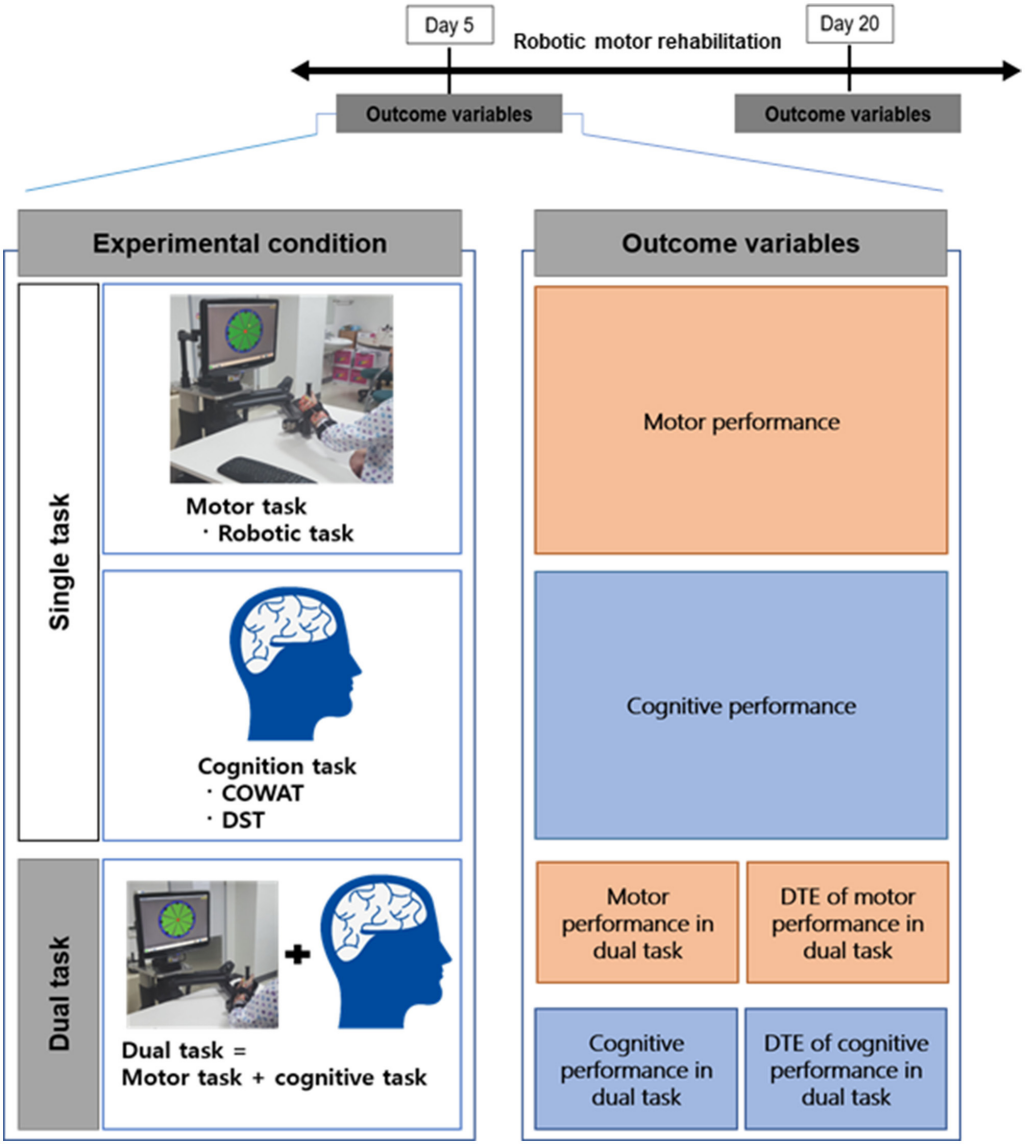
Schematic diagram of the research methodology used. Schematic diagram of the research hypothesis on capacity changes according to motor and cognitive performance. COWAT dual performance and motor performance improved from day 5 to day 20, but not for DTE. DST dual performance did not change from day 5 to day 20, only motor performance improved, but DTE did not change.

We evaluated both motor and cognitive performance, during single and dual tasks separately, on days 5 and 20 of the robotic intervention. Dual cognitive interference was performed under two conditions: (1) during the DST and (2) COWAT. The order of application of cognitive task types was randomly assigned. In the dual task condition, the participants were asked to focus on the motor task. All cognitive performances during dual tasks were recorded while the participant performed the point-to-point motor task, while motor performances during dual tasks were recorded only during the COWAT-phonemic and DSC-back tasks (Figure 1).

Dual task loss (DTL) of performance involved analyzing the effect of the cognitive task on dual-task interference and was calculated as follows: DTL (%) = [(performance in dual-task – performance in a single task)/performance in a single task] × 100% (18). For clarity, we transformed DTL into DTEs, so that higher values indicated better performance in the dual-task condition relative to the single-task condition in the following manner: DTEs of SM, MV, and cognitive performance: MV = DTL, and DTEs of RE and PE = –DTL (10).

### Statistical Analysis

We used PASW v.18.0 (SPSS Inc., Chicago, IL, USA) for statistical analysis. Descriptive statistics were used to analyze the demographic and clinical characteristics of the participants. We compared changes in cognitive or motor performance across days 5 and 20 in the single and dual tasks, respectively, using repeated-measures one-way analysis of variance (RM ANOVA). Then, repeated-measurement multivariate analysis of variance (RM MANOVA) was used to assess cognitive and motor performances during the dual task across days 5 and 20 to assess the concomitant effects of cognitive and motor tasks. Then, RM ANOVA and RM MANOVA were performed to assess the DTE of cognitive and motor performances on days 5 and 20 of robotic rehabilitation.

## Results

Thirteen stroke patients (10 males) with middle cerebral artery infarction, with a mean age of 45.9 ± 11.9 years, were enrolled in the present study. Their mean education level was 12.4 ± 4.4 years and their MMSE score was 28.2 ± 2.7.

Below, we present results for cognitive and motor performance in the context of a single task (only cognitive or motor task, without another concomitant task) and a dual task (concomitant cognitive and motor task).

### Performance in a Single Task

Table 1 demonstrates the change in motor or cognitive performance on days 5 and 20 of robotic rehabilitation. Motor performance in a single task (without a concomitant cognitive task) showed significant improvement in SM, RE, and PE, except MV.

**Table 1:** Single cognitive or motor task performance at day 5 and day 20 of robotic rehabilitation of the upper limb.

**Task performance**		**5 days**		**20 days**		**Within-subject comparisons**				
	**N**	**Mean**	**SD**	**Mean**	**SD**	**Type III sum of squares**	**df**	**Mean square**	***F***	***P***
DST-for	13	0.011	1.674	0.339	1.237	1.429	1	1.429	2.842	0.105
DST-back	13	−0.319	1.161	−0.499	1.261	0.495	1	0.495	1.017	0.323
COWAT-animal	13	−1.309	0.985	−0.736	1.154	6.169	1	6.169	17.256	<0.001
COWAT-supermarket	13	−1.282	0.795	−0.985	1.114	1.067	1	1.067	4.109	0.054
COWAT-phonemic	13	−0.422	1.247	−0.202	1.569	1.070	1	1.070	5.494	0.028
Smoothness	13	0.447	0.124	0.486	0.086	0.022	1	0.022	17.035	<0.001
Reach error	13	0.051	0.059	0.035	0.035	0.004	1	0.004	7.448	0.012
Mean velocity	13	0.098	0.049	0.098	0.038	0.000	1	0.000	0.572	0.457
Path error	13	0.025	0.029	0.018	0.013	0.001	1	0.001	5.419	0.029

*COWAT-animal, naming an animal; COWAT-supermarket, naming items in the supermarket; COWAT-phonemic: speaking with consonance “¬,” “°,” “⋏” of the Korean language, DST, digit span test, for, forward; back, backward; SD, standard deviation*.

Cognitive performance in a single task (without a concomitant motor task) demonstrated improvements in COWAT-animal, COWAT-supermarket, and COWAT-phonemic, while the DST-for and DST-back did not change.

### Performance in the Dual Task

Table 2 shows the change in motor performance between day 5 and day 20 during the dual task, in which the motor task was performed with each cognitive task (COWAT-phonemic and DST-back). RM ANOVA showed improvement in all motor performance variables except MV, in both dual tasks with COWAT and DST tasks. The COWAT performance during a concomitant motor task showed improvement, while neither DST-for or DST-back changed.

**Table 2:** Dual-task performance involving cognitive and motor performance (subdomains of cognition and motor function) at day 5 and day 20 of robotic rehabilitation of the upper limb.

				**RM ANOVA**					**RM MANOVA**				
**Task performance**		**5 days**		**20 days**		**Within-subject comparisons**					**Within-subject comparisons**				
	**N**	**Mean**	**SD**	**Mean**	**SD**	**Type III sum of squares**	**df**	**Mean square**	***F***	***P***	**Type III sum of squares**	**Df**	**Mean square**	***F***	***P***
COWAT-animal during motor task	13	−1.407	0.949	−0.602	1.099	6.169	1	6.169	17.256	<0.001					
COWAT-supermarket items during motor task	13	−1.682	0.990	−1.405	0.728	1.067	1	1.067	4.109	0.054					
COWAT-phonemic during motor task	13	−0.765	1.212	−0.442	1.436	1.070	1	1.070	5.494	0.028	1.621	1	1.621	4.212	0.063
Smoothness during COWAT-phonemic	13	0.434	0.121	0.483	0.086	0.026	1	0.026	22.970	<0.001	0.032	1	0.032	15.947	0.002
Reach error during COWAT-phonemic	13	0.056	0.054	0.044	0.041	0.003	1	0.003	4.120	0.054	0.002	1	0.002	1.428	0.255
Mean velocity during COWAT-phonemic	13	0.089	0.041	0.103	0.047	0.001	1	0.001	1.718	0.202	0.003	1	0.003	2.369	0.150
Path error during COWAT-phonemic	13	0.027	0.024	0.020	0.013	0.001	1	0.001	4.288	0.049	0.001	1	1.866	1.866	0.197
DST-for during motor task	13	0.621	1.460	1.045	1.056	1.429	1	1.429	2.842	0.105	2.159	1	2.159	2.369	0.152
DST-back during motor task	12	−0.572	0.989	−0.880	0.743	1.517	1	1.517	1.527	0.230	1.137	1	1.137	1.060	0.325
Smoothness during DST-back	12	0.432	0.122	0.477	0.095	0.022	1	0.022	17.035	<0.001	0.025	1	0.025	8.852	0.013
Reach error during DST-back	12	0.058	0.058	0.038	0.037	0.004	1	0.004	7.448	0.012	0.005	1	0.005	4.809	0.051
Mean velocity during DST-back	12	0.095	0.048	0.101	0.042	0.000	1	0.000	0.572	0.457	0.000	1	0.000	1.012	0.336
Path error during DST-back	12	0.027	0.027	0.020	0.014	0.001	1	0.001	5.419	0.029	0.001	1	0.001	3.223	1.00

*COWAT-animal, naming an animal; COWAT-supermarket, naming items in the supermarket; COWAT-phonemic: speaking with consonance “¬,” “°,” “⋏” of the Korean language, DST, digit span test; for, forward; back, backward; RM-ANOVA, repeated-measures analysis of variance; RM-MANOVA, multivariate analysis of variance; SD, standard deviation. All cognitive function scores are presented with a z-score*.

RM MANOVA was performed to examine the change in the concomitant interaction between cognition and motor performance after robotic intervention. RM MANOVA demonstrated that SM (*p* = 0.002) and COWAT-phonemic (*p* = 0.063) concomitantly improved during the dual task. In addition, there was a concomitant change in SM (*p* = 0.013), but not in the SM and DST during the dual task.

### DTEs

**Figure 2:**
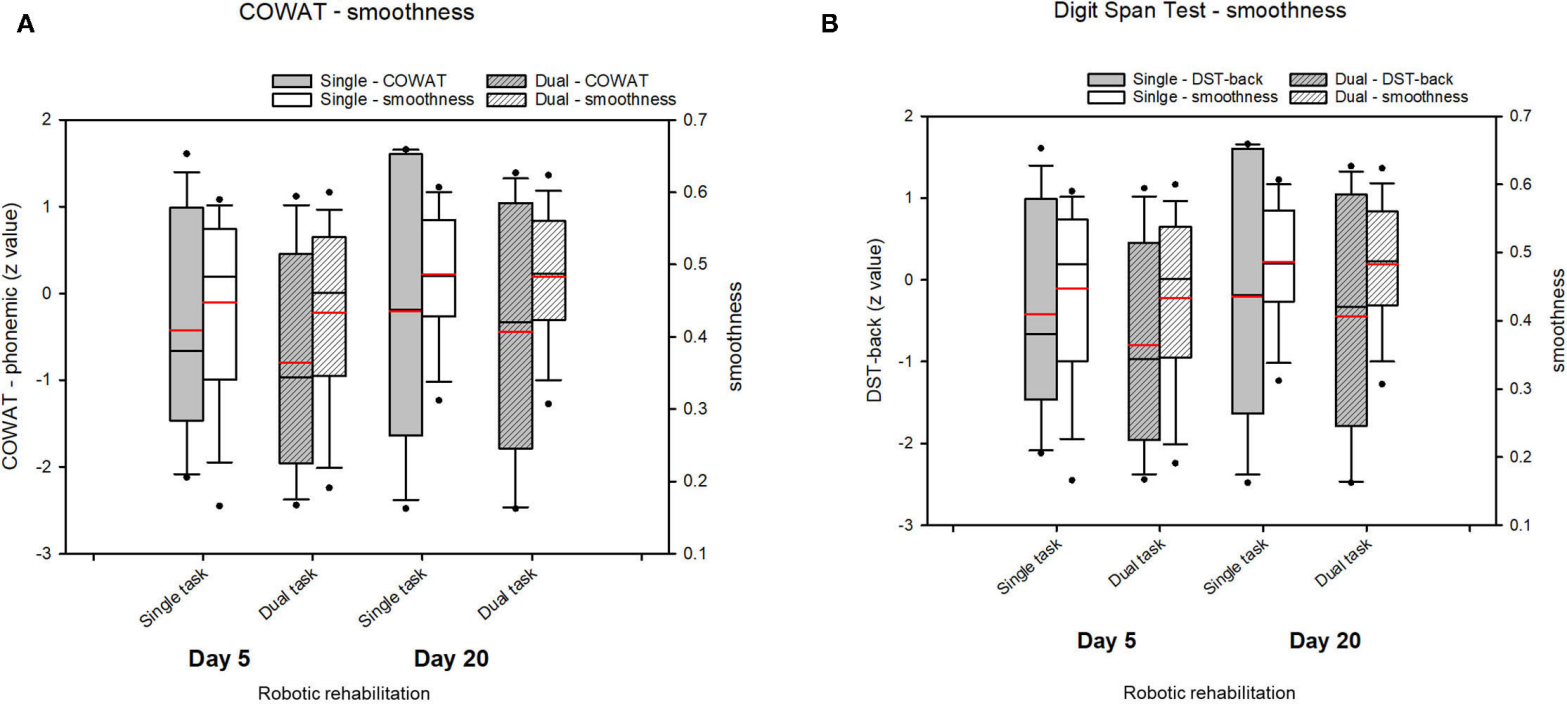
Change of the dual-task effect (DTE) value after robotic rehabilitation of the upper limbs. **(A)** COWAT_phonemic and smoothness, **(B)** Digit Span Test and smoothness. The change in the DTE of cognitive or motor performance between day 5 and day 20 during the dual task did not indicate significant changes in the DTE on motor, cognitive, or the sum of motor and cognitive performance. Boxplots represent the mean of *z*-value for each group over the course of robotic rehabilitation. Boxplots display lower and upper extremes, lower and upper quartiles, and medians. Red line in boxplots indicates the mean. The black whiskers mark the 5th and 95th percentiles.

There were no significant differences in the DTE across all cognitive tasks on day 5 (*p* > 0.300). Table 3 depicts the change in the DTE of cognitive or motor performance between day 5 and day 20 during the dual task, in which the motor task was performed with a cognitive task (COWAT-phonemic and DST-back) (Figure 2). There were no significant changes in the DTE on motor, cognitive, or the sum of motor and cognitive performance between day 5 and day 20.

**Table 3:** Dual-task effects in cognitive and motor performance at day 5 and day 20 of robotic rehabilitation of the upper limb.

**Dual-task effects during dual task**		**5 days**		**20 days**		**RM ANOVA Within-subject comparisons**					**RM MANOVA Within-subject comparisons**				
	**N**	**Mean**	**SD**	**Mean**	**SD**	**Type III sum of squares**	**df**	**Mean square**	***F***	***P***	**Type III sum of squares**	**df**	**Mean square**	***F***	***P***
DTE of DST-back	13	−1.817	5.243	−0.671	1.067	8.536	1	8.536	0.610	0.450	2.858	1	2.858	0.693	0.423
DTE of COWAT-phonemic	13	0.192	0.853	−0.059	0.577	0.378	1	0.378	1.098	0.317	0.756	1	0.756	1.098	0.317
DTE of smoothness at DST	12	−0.016	0.097	−0.016	0.089	<0.001	1	<0.001	0.000	0.993	<0.001	1	<0.001	<0.001	0.993
DTE of smoothness at COWAT-phonemic	13	−0.023	0.095	−0.004	0.062	0.002	1	0.002	0.418	0.530	0.004	1	0.004	0.418	0.530
DTE of DST-back + DTE of smoothness	12	−2.069	5.469	−0.751	1.067	2.867	1	2.867	0.690	0.424					
DTE of COWAT-phonemic + DTE of smoothness	12	0.171	0.847	−0.062	0.576	0.621	1	0.651	1.104	0.316					

*DTE, dual task effect; DST, digit span test; COWAT, Controlled Oral Word Association Test; RM-ANOVA, repeated-measures analysis of variance; RM-MANOVA, multivariate analysis of variance; SD, standard deviation*.

## Discussion

Robotic rehabilitation improved motor performance during single and dual task environments (19, 20), while cognitive performance showed different patterns of change between the DST and COWAT during single and dual tasks. However, there was no change in the DTE on motor performance, cognition performance, or the sum performance of both tasks. These results suggest that robotic rehabilitation improved performance depending on the cognitive task without altering the strategy for coping with the dual task.

We investigated changes in cognition and motor performance after robotic rehabilitation under two conditions: single-task and dual-task conditions. As our intervention involved robotic rehabilitation targeting motor function recovery rather than cognitive function, we hypothesized that performance improvement would mainly be seen in motor rather than cognitive performance regardless of single or dual task conditions. As expected, motor task performance consistently improved after robotic rehabilitation, except for mean velocity in both single and dual task conditions.

On the other hand, cognitive task performance showed a different pattern of change after robotic rehabilitation, unlike motor performance. In the single task, cognitive performance improvements were seen in every COWAT domain, but not in the DST. With the dual task, the cognitive performance in the COWAT-animal and COWAT-phonemic domains improved, while that in the COWAT-supermarket domain and DST did not. Thus, robotic rehabilitation could improve cognitive performance in some, but not all cognitive tests. These effects of robotic rehabilitation on cognitive function could be understood when considering robotic rehabilitation as a type of exercise. Exercise is known to improve multiple domains of cognitive function, but with varying effects across cognitive tasks or exercise types (21, 22). Robotic rehabilitation may have enhanced beneficial effects on cognitive function, because robotic rehabilitation places a greater attentional demand on participants to pay more attention than other exercise. Robotic rehabilitation in this study required continuous attention to the target on the screen. In addition, we inferred that robotic rehabilitation had greater effects on executive function than on working memory, as DST is related to working memory and the COWAT is more directly related to executive function (23–25).

We performed RM MANOVA using concomitant dependent variables: motor performance and COWAT-phonemic or DSC-backward was included to explore the interaction between cognitive and motor performance during robotic rehabilitation. We demonstrated significant improvement in motor performance and a marginally significant change in cognitive performance (*p* = 0.063 for the COWAT-phonemic group). Thus, robotic rehabilitation improved mainly motor, rather than cognitive performance, and these improvements were more evident during the dual-task condition. Therefore, we inferred that the task specificity of robotic rehabilitation is consistent with both dual-task and single-task conditions.

**Figure 3:**
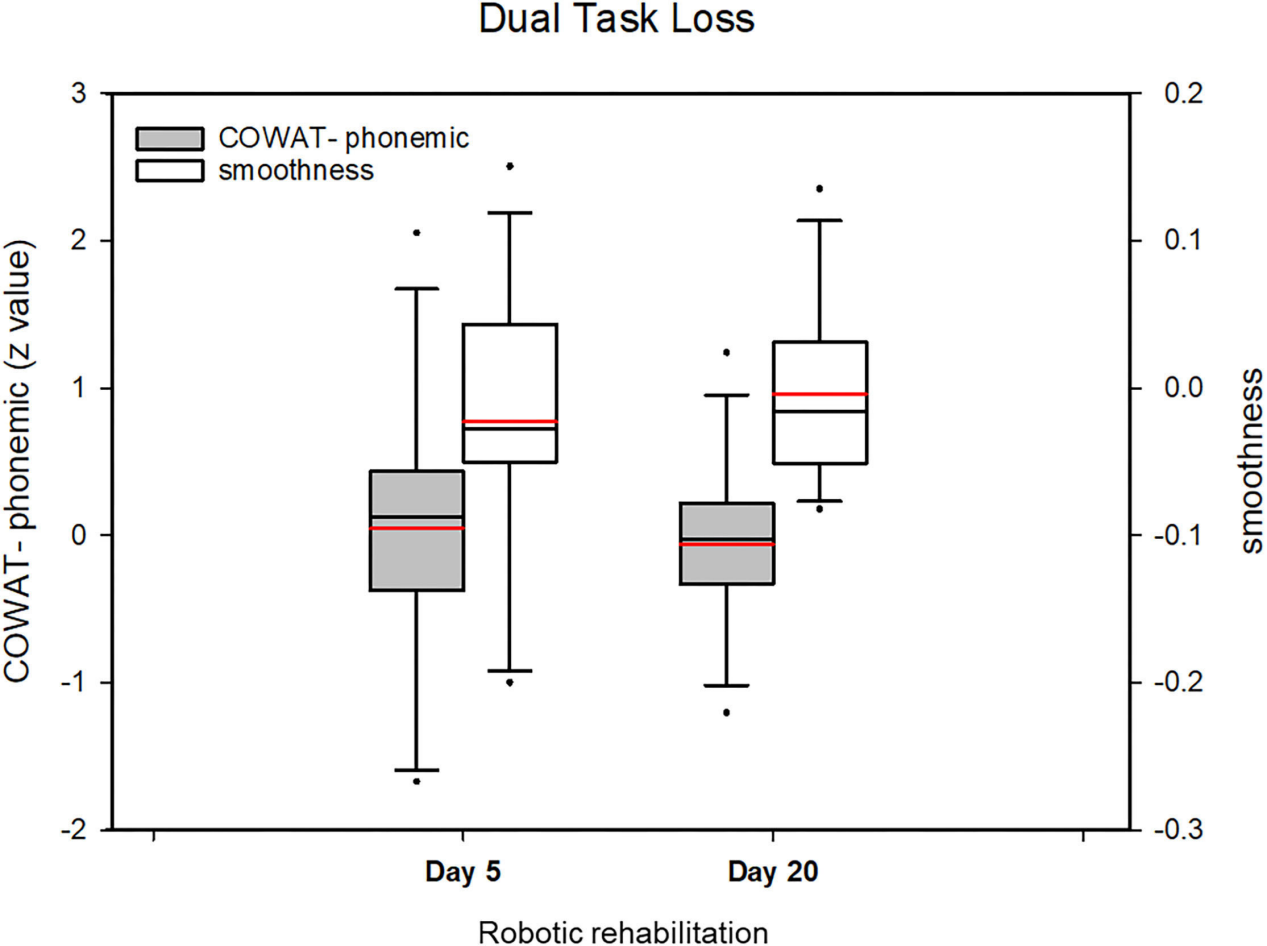
Change of the dual task loss (DTL) value (COWAT_phonemic and smoothness) after robotic rehabilitation of the upper limbs. The DTL sum for cognitive and motor function did not change. The DTL sum for cognitive and motor function did not change. Boxplots represent the mean of *z*-values for each group during the course of robotic rehabilitation. Boxplots display lower and upper extremes, lower and upper quartiles, and medians. Red line in boxplots indicates the mean. The black whiskers mark the 5th and 95th percentiles.

Next, we investigated the DTE of cognitive and motor performance (smoothness), in order to explore strategies for allocating weight between motor and cognitive tasks in dual tasks. We hypothesized that if more weight was given to the motor task, the weight allocated to the cognitive task might be reduced, or vice versa (Figure 1). In the present study, we did not find statistically significant changes in the DTE of cognitive performance and the DTE of motor performance across all cognitive tasks after robotic rehabilitation, in contrast to the improvement of performance. In addition, the DTL sum for cognitive and motor function did not change (Figure 3). Therefore, we concluded that robotic rehabilitation cannot change dual-task interference, but does affect absolute performance. This is in contrast with previous results, in which executive function training improved DTE-cognitive performance rather than DTE-motor performance (11). This difference might be explained as follows. First, our rehabilitation training was composed of point-to-point tasks that required attention as well as motor performance, thus blurring the effects of motor training effects by developing cognitive performance as well as motor performance. Second, our intervention, focusing on motor performance, might have failed to change both DTE-motor performance and DTE-cognitive performance. Interventions targeting cognitive function might easily improve DTE-cognitive performance, because cognitive function, including attention, plays an important role in controlling motor function during dual task performance (26, 27). Third, our study focused on upper extremity rehabilitation, in contrast to a previous study on gait or balance training, where participants may be injured by falling down. Therefore, the participants in our study were likely to place relatively less emphasis on motor tasks. Fourth, the DTE is known to be related to various cognitive functions; thus, the various patterns of cognitive impairment in our patients might have affected the results, in contrast to the patterns in the homogeneous older group involved in a previous study (28).

This study had several limitations. We only included a small number of participants in the single-center, which affected our results, although we achieved statistical significance. Although a normality test was not performed, statistical analysis was performed using ANCOVA analysis considering covariances by repeated measurements. The participants were stroke patients with various patterns of cognitive impairment. To overcome these limitations, we explored kinematics, using a rehabilitation robot, and only included stroke patients with right hemispheric lesions. In addition, in the statistical analyses, we adjusted for cognitive performance using standardized Z-scores according to age and educational level. Further studies in a large number of participants including a comparator group with diverse cognitive functional measurements are needed.

In the present study, modality transfer of robotic upper limb rehabilitation to cognitive performance was not consistent depending on the cognitive task. This finding could be one factor to guide the selection of optimal candidates for robotic rehabilitation; thus, patients with motor deficits might be an optimal target population. Moreover, this limited result could indicate the need for dual task robotic training that targets both motor skills and cognition as a preferred option for patients with both motor and cognitive impairments. However, it has not been confirmed; therefore, we sought to determine the usefulness of dual task robotic rehabilitation.

## Conclusions

In this study regarding stroke patients, robotic rehabilitation changed the motor performance; however, the cognitive function differed depending on the cognitive task implemented. The rehabilitation had limited effects on motor and cognitive DTEs. Robotic rehabilitation has different effects on motor and cognitive function, with more consistent effects on motor function than on cognitive function. Although motor and cognitive performance improved after robotic rehabilitation, there were no changes in the corresponding dual-task effects.

## Data Availability Statement

The raw data supporting the conclusions of this article will be made available by the authors, without undue reservation.

## Ethics Statement

The studies involving human participants were reviewed and approved by Institutional Review Board of the National Rehabilitation Center in South Korea. The patients/participants provided their written informed consent to participate in this study.

## Author Contributions

J-HS contributed to conception and design of the study and organized the database. GP conducted experiment. KL performed the statistical analysis. J-HS and KL wrote the first draft of the manuscript and wrote sections of the manuscript. All authors contributed to manuscript revision, read, and approved the submitted version.

## Funding

This study was supported by the Translational Research Center for Rehabilitation Robots, National Rehabilitation Center, Ministry of Health and Welfare, Republic of Korea (grant nos. NRCTR-IN14002 and NRCTR-IN15002).

## Conflict of Interest

The authors declare that the research was conducted in the absence of any commercial or financial relationships that could be construed as a potential conflict of interest.

## Publisher's Note

All claims expressed in this article are solely those of the authors and do not necessarily represent those of their affiliated organizations, or those of the publisher, the editors and the reviewers. Any product that may be evaluated in this article, or claim that may be made by its manufacturer, is not guaranteed or endorsed by the publisher.

## Abbreviations

CMI: Cognitive-motor interference
COWAT: Controlled Oral Word Association Test
DTE: dual-task effects
DST: Digit span test
DLT: dual task loss
MANOVA: multivariate repeated-measures analyses of variance.
